# Reliability and validity of the German version of the Forensic Restrictiveness Questionnaire

**DOI:** 10.3389/fpsyt.2025.1566694

**Published:** 2025-05-20

**Authors:** Peggy Walde, Birgit Angela Völlm

**Affiliations:** ^1^ Forensic Mental Health Research, Research Division, LVR-Institute for Research and Education, Rhineland Regional Council, Cologne, Germany; ^2^ Clinic for Forensic Psychiatry, Rostock University Medical Center, Rostock, Germany

**Keywords:** forensic psychiatry, forensic mental health, questionnaire, validity, reliability, restrictiveness, patient centered care

## Abstract

**Introduction:**

The Forensic Restrictiveness Questionnaire (FRQ) is a self-administered questionnaire for forensic mental health inpatients about their subjective experiences of restrictiveness. The present paper describes the validation of the German version of the FRQ.

**Methods:**

Patients were recruited from eight forensic psychiatric hospitals in Germany. Internal consistency was explored using Cronbach’s α. The German version of the EssenCES (assessing ward climate), the MQPLa (assessing quality of life) and the Mental Health Component subscale of the SF-12 were used to explore convergent validity. The Physical Health Component subscale of the SF-12 was used to explore divergent validity. Patient’s levels of leave (Lockerungen), substance use behavior and occurrence of incidents were used to describe criterion validity.

**Results:**

The analysis indicated very good internal consistency according to Cronbach’s α. Convergent validity could be confirmed as the FRQ-G mean score was significantly negatively correlated with the EssenCES mean score and the MQPLa mean scores. No sufficient correlation could be shown for the Mental Health Component of the SF-12. A low correlation was found with the physical component of the SF-12, indicating discriminant validity. Very few significant correlations were found to establish criterion validity.

**Discussion:**

The data indicate the FRQ-G to have good construct validity (structural, convergent, divergent) but failed to fully demonstrate criterion validity. Possible reasons include an underpowered sample size and possible measurement errors. Implications for future research are discussed.

## 1 Introduction

Forensic psychiatry is a section of psychiatry concerned with the treatment of mentally disordered or substance abusing offenders. The aim is to treat the underlying illness of the offending and thus prevent serious unlawful acts in the future (§§ 63, 64 German Penal Code). The fundamental rights of patients may be restricted for this purpose (1). The term Restrictiveness in relation to (forensic) psychiatry is often associated with involuntary treatment and the use of coercive measures, such as seclusion or restraint (2–4). Only a few authors mention the more subtle elements like a restricted number and type of personal belongings or limited contact to friends and family outside the clinic (5). In publications, a definition of this feeling of restrictiveness is often missing and, thus, left to the interpretation of the reader (e.g. (6). Definitions relating to the subjective perception of restrictiveness of patients are hard to find. (7) developed an initial approach for forensic mental health patients. He described the subjective feeling of restrictiveness as:

*“the extent to which phenomena created, maintained or augmented directly or indirectly by forensic psychiatric care are subjectively experienced by a resident as infringing negatively upon their autonomy, self or personhood”* (7, p. 253).

With this definition Tomlin (7) referred to the feeling of restrictiveness as global experience that does not depend on individual characteristics of a hospital, but rather on how patients perceive these characteristics. The decisive factor is the perceived influence on autonomy, self and experience as a human being.

Restrictive forms of accommodation were associated with various, sometimes undesirable effects. Several studies mention negative feelings like fear or anxiety (3) but also boredom and frustration (5). In addition behavioral consequences have been described, e.g., lower compliance with measures to prepare independent living and vocational training for young people living in foster care that was subjectively experienced as restrictive (8). Since restrictive elements in foster homes and forensic psychiatric hospitals have shown some overlap (e.g., restrictions regarding access to money, organization of leisure time and relationships, 5, 8, 9) it seems reasonable to assume that a strong feeling of restrictiveness also has negative effects on treatment compliance. Furthermore, perceived institutional restraint has been shown to be related to increased hostility, depression and suicidal ideation (10). These negative emotions might lead to further adverse effects. For example, increased depression rates are associated with more craving (11) and substance use relapses (12).

Therefore, being aware of the patients’ subjective feeling of restrictiveness could open opportunities for staff to support them better. As a result, therapeutic compliance could be stabilized or enhanced and incidents (e.g., hostile behavior or substance use relapse) as an expression of dysfunctional handling of negative emotions could be prevented. Furthermore, good patient support has been found to be associated with lower rates of incidents (13). Research on the feeling of restrictiveness could also help do develop psychological, nursing or other interventions further.

To examine the subjective feeling of restrictiveness in forensic mental health patients the Forensic Restrictiveness Questionnaire (FRQ, 7) was developed. It consists of a single scale without subscales with 15 statements for which patients are asked to rate the extent to which they apply to them on a 5-point Likert scale (1 = strongly disagree, 5 = strongly agree). The internal consistency has been rated as very good. Convergent validity was demonstrated using correlations of the FRQ with ward climate and quality of life. In a British sample of forensic mental health patients, Tomlin (7) found a high negative correlation between the values of the FRQ and ward climate (Schalast und Tonkin 2016) and quality of life of forensic patients (FQL-SV short version, 14). To our knowledge, no equivalent questionnaire exists to explore this feeling of restrictiveness in Germany today. Therefore, the following study aimed to validate the German translation of the FRQ for use in a German forensic mental health population.

### 1.1 Hypotheses

1. Internal consistency – According to the original FRQ, Cronbach’s alpha was expected to be around α=.90.
2. Convergent Validity was examined by the FRQ-G’s correlation with the German versions of the EssenCES, the MQPLa (Overall and subscales of a) *respectful interaction*, b) *transparency of processes and decisions* and c) *equal treatment of patients*) and the Mental Health Component subscale of the SF-12. We expected the following correlations according to the methodological literature (15):
  - The FRQ-G shows a high negative correlation with the overall EssenCES of at least r=-.50, indicating strong feelings of restrictiveness to correspond with lower values of ward climate.
  - The FRQ-G shows a high negative correlation with the overall MQPLa of at least r=-.50, indicating strong feelings of restrictiveness to correspond with lower values of quality of life.
  - The FRQ-G shows a high negative correlation with the subscales *respectful interaction* (short: Respect), *transparency of processes and decisions* (short: Transparency) and *equal treatment of patients* (short: Equality) of the MQPLa of at least r=-.50, indicating strong feelings of restrictiveness to correspond with lower values of perceived respectful interactions, lower transparency and lower perception of equal treatment.
  - The FRQ-G shows a high negative correlation with the mental component subscale of the SF-12 of at least r=-.50, indicating strong feelings of restrictiveness to correspond with lower values of physical health.
3. Discriminant validity was examined by the FRQ-G’s correlation with the Physical Health Component subscale of the SF-12. We expected a low correlation of no more than r=-.40 (15).
4. Criterion validity was examined by the FRQ-G’s correlation with patient’s levels of leave at the time the questionnaire was filled out and occurrence/frequency of substance use and certain incidents within 3 months after filling out the FRQ-G. We expected the following:
  - The FRQ-G shows a negative correlation with patient’s levels of leave of at least r=-.20, indicating strong feelings of restrictiveness to correspond with lower levels of leave.
  - The FRQ-G shows a positive correlation with patient’s frequency of substance use within 3 months after filling out the FRQ-G, according to clinical documentation and laboratory test results, of at least r=.20, i.e. strong feelings of restrictiveness correspond with more substance use.
  - The FRQ-G shows a positive correlation with occurrence and frequency of the following incidents within 3 months after filling out the FRQ-G, according to clinical documentation, of at least r=.20, indicating strong feelings of restrictiveness to correspond with more occurrence (yes/no) and higher frequencies of incidents.
    - verbal aggression against staff or fellow patients (including offensive gestures and verbal or sexual harassment).
    - physical aggression against staff or fellow patients (including sexual assaults and attempts).
    - physical aggression against objects (damage to property or vandalism, arson and attempts).

## 2 Materials and methods

### 2.1 Design, setting, and participants

This study was designed as a cross-sectional study and, for the criterion validity, a prospective cohort study. All questionnaires were scored once by the patients and, for criterion validity, hospital records were searched for the period of three months after the date of completion of the questionnaire.

The study took place in eight forensic psychiatric hospitals across Germany that provide treatment to patients under Section 63 (severe mental illness) and 64 (substance use disorder) of the German Penal Code. We included adult inpatients (from 18 years) who were detained under these sections and had been in the hospital for at least one week. All included patients were able to give informed consent, according to the multiprofessional treatment team. We included patients with a migrational background, if an acculturation to German culture was given according to the Frankfurter Akkulturationsskala (Frankfurt Acculturation Scale, 16). We also included patients with preliminary detention status who currently underwent trial (Section 126a German Code of Criminal Procedure), if the clinic expected them to become detained under one of the relevant sections. This was checked six months later to make sure that this assessment was correct. We excluded patients who did not fulfill one or more of the criteria mentioned above. We also excluded outpatients since some of the FRQ-G items would not make sense for them (e.g., “The rules on the ward are fair.”).

### 2.2 Procedure

*A priori* power analyses using G*Power 3.1 were calculated to check the required sample sizes. We set the significance level at.05 and the power to.95. For the convergent validity, the literature (e.g. 15) recommends correlations of at least.50 which resulted in a sample size of at least 38 persons. For an effect size of.40 for divergent validity, 63 persons are required and for the criterion validity with an effect size of at least.20, 266 persons are needed.

Data were collected between November 2022 and December 2023. Eligible patients were contacted by hospital staff and given an envelope with the study information and the questionnaires. Depending on the research hypothesis and the *a priori* power analysis, the numbers of questionnaires given to the patients differed. In the beginning, we gave all questionnaires to all patients. However, external clinics gave feedback that this would be overwhelming for many patients, especially for those with severe mental disorders (Section 63). Therefore, we decided to split the questionnaires in order to enhance patients’ motivation to participate. Therefore, patients received the FRQ-G together with either the rather long MQPLa or the shorter EssenCES and the SF-12. The respective questionnaires were distributed until we received the required number of analyzable questionnaires back. Patients received a compensation of 5€ for their participation. Due to the burden that a collection of the data for criterion validity would have placed on the staff of external clinics, these information were only collected from patients at the author’s clinic.

### 2.3 Material

#### 2.3.1 Quality of Life (MQPLa)

The adapted Measuring the Quality of Prison Life Questionnaire (10) is a self-report measure on quality of life in forensic mental health hospitals. The questionnaire is composed of 64 items on a 5-point Likert scale across 11 domains. We used the overall mean score and the mean scores of three subscales (*Respect*, *Transparency* and *Equal Treatment*). Higher mean scores are associated with higher rates of overall quality of life, perceived respect, transparency and equal treatment. The questionnaire has been validated in Germany within a forensic psychiatric population. Internal consistency according to Cronbach’s α was.95, with.83 for the subscale Respect,.81 for Transparency and.82 for Equal Treatment (10).

#### 2.3.2 Ward climate (EssenCES)

The patient version of the EssenCES is a self-report measure on ward climate in forensic mental health hospitals. The questionnaire is composed of 17 items on a 5-point Likert scale across three domains. Two of these items are included as fillers and not used for final calculations. We used the overall mean score for validation. Higher mean scores are indicative for a positive ward climate whereas low mean scores indicate a poor ward climate. The questionnaire has been validated in German forensic psychiatric hospitals. Internal consistency (Cronbach’s α) was between.79 and.87 on the subscales of the German patient sample (17).

#### 2.3.3 Physical and Mental Health

Physical and mental health were assessed using the SF-12, which is the short form of the Health Perception Questionnaire (SF-36; 18). We used the self-report questionnaire with a one-week time window. The SF-12 has two subscales (Physical and Mental Health Component) with six items each. We calculated the scores according to the scoring procedure suggested in the manual. The results range from zero to 100 where higher scores indicate better physical and/or mental health. The validation on a German patient sample in psychosomatic rehabilitation clinics showed an internal consistency in the acceptable range (Cronbach’s α = .70 for the mental and.78 for the physical health component scale).

#### 2.3.4 Levels of leave (“Lockerungen”)

Levels of leave describe the gradual withdrawal of security and control measures over the course of detention. This includes leave outside the clinic with or without staff and its duration (several hours up to several weeks or months). Levels of leave range from zero (unsupervised leave on the secure hospital grounds) to 10 (living outside the clinic on a trial basis). The level of leave of each patient was taken from the clinic documentation. Only patients from the clinic the authors were working at were included.

#### 2.3.5 Substance use and incidents

Occurrence and frequency of substance use were taken from clinic documentation. Again, we only looked at patients from the clinic the authors were working at. We retrieved two kind of data: first, we looked at the clinic’s documentation of incidents. This documentation also included suspicions of staff, e.g. because of suspicious behavior (e.g., slurred speech), physical appearance (e.g., very red eyes) or the discovery of drugs or related utensils in the patient’s room. Second was the documentation of positive test results, e.g., from saliva, urine or breath alcohol tests. If several positive results were documented on one day, this was counted as one event. Occurrence and frequency of incidents was taken from the same hospital documentation as the substance use. They were assigned by the authors into the following categories:

- Verbal aggression – insulting and/or threatening statements against fellow patients, staff or third parties (e.g. visitors) including corresponding gestures (e.g. outstretched middle finger) as well as sexually suggestive statements and gestures without physical contact. Statements against absent persons are included if they fulfill the above conditions.
- Physical aggression including attempts - deliberate physical harm to other people (fellow patients, staff, third parties) and sexual assaults with non-consensual physical contact.
- Damage to property, vandalism, arson, including attempts - deliberate damage or destruction of own or third-party property, including hospital property; severe soiling of objects or equipment was not included here.

We also aimed to include incidents of escape or absconsion or attempts, self-harming behavior (incl. suicide/attempts), use of coercive measures (fixation and forced medication). Due to very few or no such incidents in the relevant time period, no further such analyses were possible.

### 2.4 Data analyses

The internal consistency of the FRQ-G was determined using Cronbach’s α. Convergent validity was determined via Spearman correlations of the FRQ-G mean score with the mean scores of the overall scale of the MQPLa and its three subscales *Transparency*, *Equality* and *Respect*, as well as with the overall scale mean score of the EssenCES. Scores for the Mental and Physical Health Components of the SF-12 were calculated according to the instructions in the questionnaire’s manual. Discriminant validity was assessed using the Spearman correlation of the SF-12 Physical Health Component score and the FRQ-G mean score.

The criterion validity of the FRQ-G was determined using the Spearman correlation of the FRQ-G mean score with the occurrence (dichotomous - yes/no) and frequency of substance use and incidents. Spearman correlation was also calculated for the correlation of FRQ-G mean score and the levels of leave. All calculations were performed using SPSS 28.

## 3 Results

### 3.1 Demographic data

The original sample consisted of 184 patients. Nine patients were excluded, five of them because of missing documents (FRQ-G, signed declaration of consent), two due to incorrect legal paragraphs and two for other reasons. The final sample consisted of 175 patients. Of these, 161 (92%) were male, and 36 (20.6%) had a migration background. 62 patients were detained under Section 63 (severe mental disorder) and 113 under Section 64 (substance use disorders). One patient was preliminary detained at the time he filled out the questionnaires. This was later changed to Section 63 and we assigned him to this subsample. All patients received the FRQ. Furthermore, 88 patients returned the MQPLa, 81 the EssenCES, and 160 the SF-12. Clinical records for Lockerungen, substance use and incidents from the author’s clinic were retrieved from 87 patients.

Characteristics of the overall sample and the subsamples are depicted in **Table 1**. The number of patients recruited in each clinic is shown in **Supplementary Appendix 1**. **Table 2** shows an overview of the descriptive characteristics of the questionnaires.

**Table 1:** Characteristics of the overall sample and the samples for each questionnaire and the Lockerungen/incidents retrieved from the clinical documentation.

	FRQ-G/Overall sample	MQPLa	EssenCES	SF-12	Lockerungen/Incidents
N	175	88	81	160	87
Age	*M* = 35.21 *SD* = 9.480	*M* = 36.02 *SD* = 10.032	*M* = 34.89 *SD* = 9.576	*M* = 35.32 *SD* = 9.438	*M* = 33.59 *SD* = 8.410
Legal section					
• 63	62 (35.4%)	40 (45.5%)	28 (34.6%)	54 (33.3%)	10 (11.5%)
• 64	113 (64.6%)	48 (54.5%)	53 (65.4%)	108 (66.7%)	77 (88.5%)
Sex					
• Male	161 (92.0%)	82 (93.2%)	70 (86.4%)	153 (94.4%)	81 (93.1%)
• Female	12 (6.9%)	6 (6.8%)	9 (11.1%)	9 (5.6%)	6 (6.9%)
• Other	2 (1.1%)	0	2 (2.5%)	0	0
Diagnosis^1^					
• F1	127 (72.6%)	65 (73.9%)	63 (77.8%)	121 (74.7%)	82 (94.3%)
• F2	30 (17.1%)	18 (20.5%)	15 (18.5%)	26 (16.0%)	11 (12.6%)
• F6	53 (30.3%)	29 (33.0%)	25 (30.9%)	49 (30.2%)	24 (27.6%)
• F7	20 (11.4%)	18 (20.5%)	10 (12.3%)	20 (12.3%)	10 (11.5%)
• Other	43 (24.6%)	25 (28.4%)	23 (28.4%)	41 (25.3%)	27 (31.0%)

^1^ According to ICD-10 chapters: F1 = Substance use disorders, F2 = Schizophrenia and other delusional disorders, F6 = Personality Disorders incl. Paraphilia, F7 = Intellectual Disabilities; multiple diagnosis possible.

**Table 2:** Descriptive statistics of questionnaire measures.

	FRQ-G*	MQPLa	MQPLa Respect*	MQPLa Equal.	MQPLa Transp.	Essen-CES*	SF-12 mental*	SF-12 physical*
N	175	88	88	88	88	81	120	120
Mean	2.37	2.51	2.54	2.09	2.34	2.23	44.98	49.30
SD	0.763	0.551	0.730	0.960	0.727	0.630	11.815	7.972
Median	2.333	2.615	2.667	2.200	2.429	2.333	48.113	52.000
Range	1.00-4.40	1.31-3.84	0.50-4.00	0.00-4.00	0.43-4.00	0.67-3.40	11.94-63.89	22.50-62.50

Statistics for overall mean scores (FRQ-G, MQPLa overall and subscales, EssenCES) and calculated SF-12 subscale scores.

SD, Standard deviation.

*Shapiro-Wilk test significant with p <.05, normal distribution not given.

### 3.2 Internal consistency

The internal consistency of the FRQ-G (with 15 items) was calculated using Cronbach’s α for the total sample and for both subsamples. Only complete questionnaires (N=157) were included in the analysis. The internal consistency was high, with Cronbach’s α = .88 in the overall sample. Similar values were obtained for the subsamples with equally high internal consistencies with Cronbach’s α = .88 (Section 63) and = .87 (Section 64). The item discrimination index for each individual item was above.30, meaning that none of the items should be discarded (15). The results are shown in Appendix II.

### 3.3 Convergent and discriminant validity

The FRQ-G correlated significantly negative with the EssenCES and the MQPLa total score as well as the three subscales examined. All correlations except for the EssenCES in the Section 64 subsample are at least r =-.50 thus fulfilling the minimum values described in the literature and confirm the hypothesis. However, the correlations did not reach the values from the British validation study. In contrast, the hypothesis could not be confirmed for the Mental Health Component Scale of the SF-12, which only demonstrated a negative (yet significant) correlation below the required minimum.

Discriminant validity was assessed using the Physical Health Component Scale of the SF-12. The correlation with the FRQ-G was below r = -.40, in line with the hypothesis indicating discriminant validity is given. The results of the subsamples were similar to those of the overall sample. All results are shown in **Table 3**.

**Table 3:** Convergent and discriminant validity of the FRQ-G with the overall and subsamples.

	Overall Sample	Section 63	Section 64
	r (p)	r (p)	r (p)
EssenCES	-.625 (<.000)	-.762 (<.000)	-.403 (.003)
MQPLa – Overall	-.721 (<.000)	-.743 (<.000)	-.777 (<.000)
MQPLa – Respect	-.645 (<.000)	-.625 (<.000)	-.674 (<.000)
MQPLa – Transparency	-.586 (<.000)	-.549 (<.000)	-.687 (<.000)
MQPLa – Equal Treatment	-.560 (<.000)	-.734 (<.000)	-.500 (<.000)
SF-12 – Mental Health	-.226 (.005)	-.264 (.064)	-.207 (.037)
SF-12 – Physical Health	-.163 (.044)	-.088 (.543)	-.192 (.053)

r, Spearman correlation; p, p-value.

### 3.4 Criterion validity

Criterion validity was examined using the correlation of the FRQ-G mean value with the patient’s level of leave, and the occurrence and frequency of substance use and other incidents within 3 months after completing the FRQ-G. An overview of the distribution of the levels of leave and the number of patients with substance use or incidents is shown in **Tables 4** and **5**. Most of the patients had levels of leave below 6, meaning they were not yet allowed to leave the clinic alone for more than six hours per day or stay outside the clinic overnight.

**Table 4:** Distribution of levels of leave in the overall sample and the subsamples when filling out the FRQ-G.

	Overall sample	Section 63	Section 64
N	87	n = 10 (11.5%)	n = 77 (88.5%)
Levels of leave			
• 0 (no leave outside clinic)	26 (29.9%)	4 (40.0%)	22 (28.6%)
• 1	17 (19.5%)	2 (20.0%)	15 (19.5%)
• 2	12 (13.8%)	0 (0.0%)	12 (15.6%)
• 3	1 (1.1%)	0 (0.0%)	1 (1.3%)
• 4	14 (16.1%)	3 (30.0%)	11 (14.3%)
• 5	2 (2.3%)	0 (0.0%)	2 (2.6%)
• 6	7 (8. 0%)	1 (10.0%)	6 (7.8%)
• 7	3 (1.7%)	0 (0.0%)	3 (3.9%)
• 8	2 (1.1%)	0 (0.0%)	2 (2.6%)
• 9	2 (1.1%)	0 (0.0%)	2 (2.6%)
• 10	1 (0.6%)	0 (0.0%)	1 (1.3%)

**Table 5:** Number of patients causing incidents within 3 months after completing the FRQ-G.

	Overall Sample	Section 63	Section 64
Total number of patients	N = 87	N = 10	N=77
Incidents			
• Verbal aggression	18 (20.7%)	4 (40.0%)	14 (18.2%)
• Physical aggression^1^	3 (3.4%)	0 (0.0%)	3 (3.9%)
• Damage to property, vandalism, arson^1^	6 (6.9%)	3 (30.0%)	3 (3.9%)
Substance Use			
• Documentation	16 (18.4%)	0 (0.0%)	16 (20.8%)
• Test results (missing: 1)	26 (29.9%)	1 (10.0%)	25 (32.9%)
Overall			
• Number of patients causing incidents	20 (23.0%)	5 (50.0%)	15 (19.5%)
• Number of incidents across all patients	68	22	46

^1^ including attempt.

As shown in **Table 5**, several incidents occurred within 3 months after completing the FRQ-G. From the 87 patients, 20 caused one or more incidents (= 23% of the overall sample). In most cases they showed verbal aggression against fellow patients or staff. Overall, 68 incidents were caused by these 20 patients. Moreover, several patients were suspected of consuming alcohol or illicit drugs (16 or 26 depending on measure).

The results of the criterion validity exploration can be found in **Table 6**. No correlations with the FRQ-G mean score could be found for levels of leave (Lockerungen). Instead, the FRQ-G showed small correlation with the overall number of incidents across all patients in the overall sample and the Section 64 subsample. This applied especially for verbal aggressions indicating high feelings of restrictiveness to be associated with higher numbers of incidents and especially a higher number of verbal aggression. Negative correlations between FRQ-G mean scores and property damage were found in the overall sample, indicating feelings of restrictiveness to be associated with less property damage (which contradicts the hypothesis). No other correlations for incidents were significant. On the other hand, significant correlations could be found between the FRQ-G mean score and the number of positive drug tests in the Section 64 subsample.

**Table 6:** Spearman correlations and p-values of levels of leave, incidents and substance use with FRQ-G mean score in the overall sample and subsamples.

	Overall Sample	Section 63	Section 64
N	87	10	77
Lockerungen			
	0.04 (0.737)	-0.25 (0.478)	0.08 (0.505)
Incidents			
Overall number of incidents	0.213 (0.024)	-0.07 (0.429)	0.28 (0.007)
Verbal aggression			
Occurrence	0.27 (0.007)	0.28 (0.213)	0.27 (0.009)
Frequency	0.24 (0.014)	0.15 (0.339)	0.25 (0.013)
Physical aggression^1^			
Occurrence	-0.05 (0.314)	^2^	-0.06 (0.310)
Frequency	-0.05 (0.308)	^2^	-0.06 (0.303)
Property Damage^1^			
Occurrence	-0.20 (0.031)	-0.50 (0.073)	-0.11 (0.170)
Frequency	-0.20 (0.031)	-0.45 (0.096)	-0.11 (0.166)
Substance Use			
Documentation			
Occurrence	0.05 (0.332)	^2^	0.06 (0.300)
Frequency	0.05 (0.309)	^2^	0.07 (0.276)
Test			
Occurrence	0.17 (0.061)	-0.17 (0.315)	0.219 (0.028)
Frequency	0.17 (0.055)	-0.17 (0.315)	0.219 (0.028)

^1^including attempt, ^2^no incidents.

## 4 Discussion

The present paper aimed to describe parts of the validation of the German version of the Forensic Restrictiveness Questionnaire (7), the FRQ-G. In total, 184 forensic mental health patients participated. Of these, 157 returned FRQ-G questionnaires without missing data that were used to test internal consistency. Convergent and divergent validity could be tested with 88 MQPLa, 81 EssenCES and 120 SF-12 questionnaires which made this analyses sufficiently powered. Additionally, we collected data from 87 patients’ clinical records to establish criterion validity, which was not enough for sufficient power.

The results show high internal consistency comparable to that of the original questionnaire. The FRQ-G showed convergent validity according to high negative correlations with the EssenCES Questionnaire on ward atmosphere and quality of life according to the MQPLa and three of its subscales. An exception was the slightly lower correlation of the FRQ-G with the EssenCES in the Section 64 subsample. This is below the required value of at least.50 found in methodological literature (15). Patients treated under Section 64 committed their offence in relation to a substance use disorder. A legislation like this is internationally rare and not established in the UK, where the FRQ-G was developed. Therefore, further validation research on the FRQ-G and its applicability for offenders under this section in Germany is needed. Another unacceptably low correlation was found with the Mental Health Component of the SF-12 questionnaire for the overall samples and subsamples. A questionnaire like the SF-12 was not used in the original validation study. It might be possible that our theoretical derivation left out other relevant influential factors. More research is needed to describe the complex psychological relations behind the feeling of restrictiveness and its impact on patient’s physical and mental health. In terms of divergent validity, only a small correlation was found between the FRQ-G and the Physical Health Component of the SF-12. Given that, we conclude that our results indicate that convergent and divergent validity is given. Further research is needed to explore the relationship with feelings of restrictiveness and subjective reported mental health.

Not all hypotheses regarding criterion validity could be confirmed. We found indications that higher feelings of restrictiveness are associated with occurrence of more critical incidents. Moreover, higher feelings of restrictiveness were associated with more verbal aggression, which is in line with our hypothesis. Other hypothesis could not be confirmed. Some correlations even went in the opposite direction to that hypothesized, indicating that strong feelings of restrictiveness might be more related with withdrawal behavior than with acting out especially for patients with severe mental disorders. This correlation failed to reach significance but medium effect size in a clearly underpowered sample makes it reasonable that something has been overlooked. Future studies could address that. Another study in German forensic psychiatric hospitals also found a relation between perceived institutional restraint and depression (19) which could be an explanation for these findings. The same study also found an increase in hostility when patients perceived institutional restraint as high. This finding is in line with the significantly increased occurrence of verbal aggression in our sample. In addition, at least for the subsample of Section 64 patients, there are indications that strong feelings of restrictiveness lead to increased substance use according to test results. As a conclusion, we can state that the FRQ-G needs further investigation regarding criterion validity. We found indications for that but failed to fully prove it.

Moreover, we recommend further research in the construct of restrictiveness itself. The high correlation of the FRQ-G with MQPLa and EssenCES indicates at least a close relatedness of these constructs, if not representing parts of it. In fact, some of the FRQ-G items are very similar to the items of both of these questionnaires. Therefore, further investigation and delimitation of the construct appears to make sense. Nonetheless, in practical terms, the FRQ-G has clear advantages. It is a short and economic questionnaire, especially compared to the 64-item MQPLa, which clinics feedbacked to us was overwhelming for some patients. This indicated better user friendliness especially for patients with intellectual disabilities or cognitive difficulties (e.g., regarding concentration and attention control). Furthermore, its shortness might make it less likely that patients rush through it and fail to read all items carefully. Hence, it could be an interesting tool for research (e.g., in terms of health care research and as an outcome for intervention studies) and clinical routine measurement (e.g., in terms of treatment evaluation and incident prevention) if validity can be established.

### 4.1 Limitations

There are some methodological limitations for the results. First, the patients in the sample were recruited in eight different clinics in six different federal states in Germany. Recruiting was done by the staff of the clinics. Only in the clinic of the authors we did the recruitment ourselves. Despite all clinics being given the same written information about inclusion and exclusion criteria, it is not possible to say if all of the patients that fulfilled these criteria were addressed. Furthermore, we had no control on how these patients were addressed. Differences in handling could explain the remarkable differences in return rates (ranging from four returns in one clinic to 87 returns in another one). Therefore, selection bias with an over- or underestimation of the feeling of restrictiveness might be possible. Additional biases could have occurred due to differences in understanding the questionnaires. We made the experience ourselves and got feedback from the participating clinics that many patients were not used to participate in studies and are not used to read a lot. Therefore, receiving the envelope with several pages of text might have been overwhelming for some patients and kept them from participating. Other patients filled out the questionnaires with the help of clinical staff, which might opened some space for socially desired answering. These issues applied predominantly for patients with severe mental disorder detained under Section 63 and probably contributed to the low number of participants. Especially for criterion validity, the required sample size (according to the *a priori* power analysis) could not be reached. This could be an explanation for the lack of significance despite medium range effect sizes. Further research with adequate powered sample sizes is required to obtain clearer results. Furthermore, we did not use any adjustment to multiple testing which increases the probability of incorrect results. This makes our findings preliminary and replication is needed. Finally, some error variance in the results on substance use might have been caused by the way it was measured. The laboratory testing included results of saliva quick tests. It turned out that these tests had an increased false positive rate especially for amphetamines. Therefore, an overestimation of substance use in the laboratory test results is possible. On the other hand, the clinical documentation based on staffs evaluation is also prone to errors since there did not exist standardized procedures as to which suspicious behavior is to be documented and when. More standardized and objective procedures and the additional use of reliable tests might deliver a clearer picture in future studies.

## Data Availability

The raw data supporting the conclusions of this article will be made available by the authors, without undue reservation.

## References

[1] Valenti E Barrios F Luis F. Mental health and human rights in forensic psychiatry in the european union. In: Sadoff RL Baird JA, editors. Ethical issues in forensic psychiatry. Minimizing harm. Wiley-Blackwell, Chichester (2011). p. 35–55.

[2] Lawrence D Bagshaw R Stubbings D Watt A. Restrictive practices in adult secure mental health services: A scoping review. Int J Forensic Ment Health. (2022) 21:68–88. doi: 10.1080/14999013.2021.1887978

[3] Hui A. Least Restrictive Practices: An evaluation of patient experiences. Nottingham (2017). Available at: https://nottingham-repository.worktribe.com/output/900983.zuletztgeprüftam15.03.2025 (Accessed March 15 2025).

[4] Steinert T Hirsch S. S3-Leitlinie Verhinderung von Zwang: Prävention und Therapie aggressiven Verhaltens bei Erwachsenen. Berlin, Heidelberg: Springer Berlin Heidelberg (2019). 10.1007/s00115-019-00801-2 31473766

[5] Tomlin J Egan V Bartlett P Völlm B. What do patients find restrictive about forensic mental health services? A qualitative study. Int J Forensic Ment Health. (2020) 19:44–56. doi: 10.1080/14999013.2019.1623955

[6] Repo-Tiihonen E Vuorio O Koivisto H Paavola P Hakola P. Opinions about treatment modalities among patients involuntarily committed to a forensic psychiatric hospital in Finland. J Offender Rehabil. (2004) 38:81–95. doi: 10.1300/J076v38n03_06

[7] Tomlin J. Measuring Experiences of Restrictiveness in Forensic Psychiatric Care: Developing a Questionnaire. Nottingham: University of Nottingham (2019).

[8] Schmidt JD. Assessing the Impact of Restrictiveness and Placement Type on Transition-Related Outcomes for Youth With and Without Disabilities Aging Out of Foster Care. Portland. Department of Social Work: Portland State University (2015).

[9] Craik C Wendy B Amanda R Samantha B Nicole B Mason A. A qualitative study of service user experiences of occupation in forensic mental health. Aust Occup Ther J. (2010) 57:339–44. doi: 10.1111/j.1440-1630.2010.00857.x 20868423

[10] Büsselmann M Titze L Lutz M Dudeck M Streb J. Measuring the quality of life in forensic psychiatric hospitals. Front Psychol. (2021) 12:701231. doi: 10.3389/fpsyg.2021.701231 34305762 PMC8299050

[11] Darharaj M Hekmati I Farahnaz MGA Ahmadi A Eskin M Abdollahpour Ranjbar H. Emotional dysregulation and craving in patients with substance use disorder: the mediating role of psychological distress. Int J Ment Health Addict. (2024) 22:2997–3012. doi: 10.1007/s11469-023-01031-z

[12] Bradizza CM Stasiewicz PR Paas ND. Relapse to alcohol and drug use among individuals diagnosed with co-occurring mental health and substance use disorders: a review. Clin Psychol Rev. (2006) 26:162–78. doi: 10.1016/j.cpr.2005.11.005 16406196

[13] Ros N van der Helm P Wissink I Stams G-J Schaftenaar P. Institutional climate and aggression in a secure psychiatric setting. J Forensic Psychiatry Psychol. (2013) 24:713–27. doi: 10.1080/14789949.2013.848460

[14] Schel SHH Bouman YHA Vorstenbosch ECW Bulten BH. Development of the forensic inpatient quality of life questionnaire: short version (FQL-SV). Qual Life Res. (2017) 26:1153–61. doi: 10.1007/s11136-016-1461-9 27878427

[15] Bühner M. Einführung in die Test- und Fragebogenkonstruktion. 4., korrigierte und erweiterte Auflage. München: Pearson (2021). (ps Psychologie).

[16] Bongard S Etzler S Frankenberg E. FRAKK. Frankfurter Akkulturationsskala. Göttingen: Hogrefe (2020).

[17] Schalast N Tonkin M(. The Essen Climate Evaluation Schema EssenCES. A manual and more. Boston: Hogrefe Publishing Corporation (2016).

[18] Morfeld M Kirchberger I Bullinger M. SF-36 Fragebogen zum Gesundheitszustand: Deutsche Version des Short Form-36 Health Survey. Göttingen: Hogrefe (2011).

[19] Franke I Büsselmann M Streb J Dudeck M. Perceived institutional restraint is associated with psychological distress in forensic psychiatric inpatients. Front Psychiatry. (2019) 10:410. doi: 10.3389/fpsyt.2019.00410 31244698 PMC6580144

