# Supplementary material for: Reliability and validity of the German version of the Forensic Restrictiveness Questionnaire
Source: Front Psychiatry. 2025 May 20;16:1566694. doi: 10.3389/fpsyt.2025.1566694 (PMC12129968; doi:10.3389/fpsyt.2025.1566694)
Supplement: Supplementary file 1 [file Table1.docx]

Appendix

| Appendix I. Number of patients recruited per clinic. | | |
| --- | --- | --- |
|  | N | % |
| Clinic 1 | 87 | 49,7 |
| Clinic 2 | 4 | 2,3 |
| Clinic 3 | 12 | 6,9 |
| Clinic 4 | 17 | 9,7 |
| Clinic 5 | 14 | 8,0 |
| Clinic 6 | 12 | 6,9 |
| Clinic 7 | 19 | 10,9 |
| Clinic 8 | 10 | 5,7 |
| *Note.* For reasons of data protection the specific name of the institution is not mentioned. | | |

| Appendix II. Item discrimination index of the FRQ-G’s items in the overall sample and the subsamples of section 63 patients (severe mental illness) and section 64 patients (substance use disorders) | | | |
| --- | --- | --- | --- |
|  | **Overall sample** | **Section 63** | **Section 64** |
|  | N=157 | n=55 | n=102 |
| 1. Ich werde hier wie ein Mensch behandelt | .669 | .639 | .675 |
| 2. Ich habe hier genügend Möglichkeiten. meine Gefühle auszudrücken. wenn ich es möchte | .667 | .677 | .635 |
| 3. Die Klinik hilft mir dabei. Freizeitaktivitäten nachzugehen. die mir gefallen | .649 | .762 | .567 |
| 4. Ich werde genügend über meine Behandlung informiert | .537 | .524 | .549 |
| 5. Ich fühle mich genügend in meine Behandlung einbezogen (Behandlungsplanung und Visiten) | .580 | .579 | .645 |
| 6. Das Personal respektiert mich als Persönlichkeit mit all meinen Eigenschaften | .615 | .669 | .542 |
| 7. Mir wird genügend Verantwortung auf Station gegeben | .558 | .546 | .564 |
| 8. Das Personal vertraut mir genügend | .563 | .519 | .574 |
| 9. Ich kann jeden Tag entscheiden. was ich tun möchte | .575 | .558 | .582 |
| 10. Es ist gerecht. dass ich zurzeit hier bin | .524 | .504 | .508 |
| 11. Ich kann an Aktivitäten teilnehmen. die mir wichtig sind | .724 | .809 | .640 |
| 12. Meine Rechte werden hier angemessen respektiert | .703 | .701 | .688 |
| 13. Ich werde gezwungen Dinge zu tun. die ich nicht tun will | -.437 | -.451 | -.363 |
| 14. Die Regeln auf Station sind fair | .607 | .677 | .534 |
| 15. Die Einschränkungen auf Station sind sinnvoll | .642 | .665 | .615 |
